# Retrospective analysis of the overt proteinuria diabetic kidney disease in the treatmentof modified Shenzhuo formula for 2 years: Erratum

**DOI:** 10.1097/MD.0000000000007117

**Published:** 2017-06-02

In the article, “Retrospective analysis of the overt proteinuria diabetic kidney disease in the treatmentof modified Shenzhuo formula for 2 years”,^[1]^ which appeared in Volume 96, Issue 12 of *Medicine*, the authors’ affiliations appeared incorrectly. Dr. Hongdong Chen is affiliated with both the Department of Endocrinology, Guang’anmen Hospital of China, Academy of Chinese Medical Sciences and the Department of Endocrinology, Beijing Hepingli Hospital. Dr. Jing Guo is affiliated with the Department of Endocrinology, Guang’anmen Hospital of China, Academy of Chinese Medical Sciences. Dr. Zhongchen He is affiliated with the Department of Endocrinology, Beijing Hepingli Hospital. Dr. Xiaolin Tong is affiliated with the Department of Endocrinology, Guang’anmen Hospital of China, Academy of Chinese Medical Sciences.
